# Improving nutritional status among urban poor children in sub‐Saharan Africa: An evidence‐informed Delphi‐based consultation

**DOI:** 10.1111/mcn.13099

**Published:** 2020-11-03

**Authors:** Maurice Mutisya, Oonagh Markey, Emily K. Rousham, Jesman M. N. Chintsanya, Rebecca Pradeilles, Elizabeth W. Kimani‐Murage, Nyovani J. Madise, Alister C. Munthali, Alexander Kalimbira, Michelle Holdsworth, Paula L. Griffiths, Emma Haycraft

**Affiliations:** ^1^ African Population and Health Research Center Nairobi Kenya; ^2^ School of Sport, Exercise and Health Sciences Loughborough University Loughborough UK; ^3^ Department of Population Studies, Chancellor College University of Malawi Zomba Malawi; ^4^ School of Health and Related Research University of Sheffield Sheffield UK; ^5^ Wellcome Trust London UK; ^6^ African Institute for Development Policy Lilongwe Malawi; ^7^ Centre for Social Research, Chancellor College University of Malawi Zomba Malawi; ^8^ Lilongwe University of Agriculture and Natural Resources Lilongwe Malawi; ^9^ UMR NUTRIPASS, Research Institute for Development Montpellier France

**Keywords:** complementary feeding, growth, infant nutrition, LMIC, stunting, sub‐Saharan Africa, undernutrition

## Abstract

In sub‐Saharan Africa (SSA), rapid urbanisation coupled with the high prevalence of infant and young child (IYC) undernutrition in low‐income settings means that interventions to support IYC nutrition are a priority. Little is known about how urbanisation influences IYC feeding (IYCF) practices, and evidence‐based interventions to improve IYC health/nutrition in the urban poor are lacking. Therefore, this research aimed to (a) systematically review evidence on interventions for improving the nutritional status of IYC aged 6–23 months living in urban poor areas (PROSPERO CRD42018091265) and (b) engage stakeholders to identify the highest ranking evidence gaps for improving IYCF programmes/policies. First, a rapid systematic review was conducted. This focused on the literature published regarding nutrition‐specific and nutrition‐sensitive complementary feeding interventions in urban poor areas, specifically low‐income informal settlements, in low‐ and middle‐income countries (LMICs). Six intervention studies met the review inclusion criteria. Intervention adherence was generally high, and indicators of maternal knowledge and IYC nutritional intake typically increased because of the interventions, but the impact on anthropometric status was small. Second, stakeholders working across SSA were engaged via a Delphi‐based approach to identify priority areas for future intervention. Stakeholders reported that a situational analysis was required to better understand IYCF in urban poor areas, particularly the causes of IYC undernutrition, and highlighted the need to involve local communities in defining how future work should proceed. Together, these findings indicate a need for more evidence regarding IYCF and the factors that drive it in urban poor areas across LMIC settings, but particularly in SSA.

**Key messages**

- Intervention studies to improve nutritional status in infants and young children (IYC) living in urban low‐income informal settlements or slums in low‐ and middle‐income countries, including sub‐Saharan Africa (SSA), have focused primarily on outcomes related to anthropometric status.
- Future complementary feeding interventions should include an assessment of diet quality.
- Nutrition‐sensitive factors should be considered when developing and evaluating complementary feeding interventions in urban low‐income informal settlements or slums; this will help to maximise the potential impact and effectiveness of nutrition‐specific interventions.
- Multisectoral approaches are needed to improve the nutritional status of urban poor children of complementary feeding age in SSA.

## 1 INTRODUCTION

Stunting, caused by long‐term undernutrition, is a significant global health concern (UNICEF, 2020; World Health Organization [WHO], 2012). Stunted children experience impaired growth and development with lifelong consequences on morbidity and mortality (Black et al., 2008). Suboptimal complementary feeding, including inappropriate timing of introduction of semi‐solid and solid foods, is associated with stunting in low‐ and middle‐income countries (LMICs) (Marriott, White, Hadden, Davies, & Wallingford, 2012). Currently, less than one in five children in low‐ and lower middle‐income countries receive a minimum acceptable diet (MAD; a composite indicator composed of minimum meal frequency [MMF] and minimum dietary diversity [MDD]) (UNICEF, 2019). Across sub‐Saharan Africa (SSA), the proportion of children receiving the MAD during complementary feeding has been low and is declining. For instance, Demographic and Health Survey (DHS) data (2010–2013) from 10 SSA countries revealed that less than 18% of 6–23‐month‐olds met the MAD (Na, Jennings, Talegawkar, & Ahmed, 2015). Between 2008 and 2014, the proportion of 6–23‐month‐olds who received the MAD decreased from 39% to 22% in Kenya (Kenya National Bureau of Statistics [KNBS], 2015) and from 19% to 8% in Malawi (National Statistical Office, 2011; National Statistical Office, 2017), indicating a significant worsening of diet. In Tanzania, DHS data showed a decline of both MDD and MAD between 2004/2005 and 2015/2016 in 6–23‐month‐olds from 46% to 30% and 16.6% to 6% respectively, while MMF remained constant (Ogbo, Ogeleka, & Awosemo, 2018).

Poverty and poor living conditions in LMICs are also associated with stunting. In 2019, two out of five stunted infants and young children (IYCs) globally lived in SSA, which is significantly higher than the global average of one in five (21.3%) children under 5 years who are stunted (UNICEF, 2020). Children living in slums are more likely to experience undernutrition than other urban children (Ahsan, Arifeen, Al‐Mamun, Khan, & Chakraborty, 2017; Unger, 2013). SSA is experiencing rapid urbanisation and nutrition transition with consequent changes in the social and physical environments and shifts in food habits and practices. By 2050, the African continent will have 1.5 billion urban dwellers, equal to 22% of the world's urban population (United Nations [UN], 2019). The urban population in SSA is increasing through high rates of rural‐to‐urban migration, natural population growth and the reclassification of towns to urban centres. The spread of urban low‐income informal settlements and undernutrition have increased alongside rapid urbanisation (Awumbila, 2017; Fotso et al., 2012; Nickanor & Kazembe, 2016). However, less is known about how urbanisation influences IYCF practices, particularly around complementary feeding, and evidence‐based interventions to improve IYC health/nutrition in slums are lacking (Ezeh et al., 2016; Goudet, Bogin, Madise, & Griffiths, 2019; Lilford et al., 2016).

It is estimated that interventions to promote optimal IYCF practices could prevent 20% of deaths in under 5 years in countries with high child mortality (Alderman, Nguyen, & Menon, 2019; Jones et al., 2003; Kramer & Kakuma, 2004, 2012). Optimal feeding practices include breastfeeding initiated within 1 h of birth, exclusive breastfeeding for 6 months after birth, and introducing safe, nutritionally adequate complementary solid food at 6 months (alongside continued breastfeeding up to 24 months or longer) (WHO, 2020). Current approaches to improve nutritional status include both *nutrition‐sensitive interventions*, which address the underlying determinants of nutrition (e.g., agriculture and food security, early child development, maternal mental health, health and family planning services, and water, sanitation and hygiene [WASH]), and *nutrition‐specific interventions*, which address proximal determinants of nutrition (e.g., adequate food and nutrient intake, feeding, caregiving and parenting practices, and low burden of infectious diseases) (Ruel et al., 2013).

A Cochrane systematic review of nutritional interventions for preventing stunting in children under 5 years in poor urban areas in LMICs showed that nutrition‐specific interventions have the potential to decrease stunting in nonslum contexts, but this efficacy is inconsistent when these interventions are applied in urban slums (Goudet et al., 2019). This finding was attributed to environmental challenges associated with conducting interventions in these settings, including the lack of social services, high mobility and attrition rates (Goudet et al., 2019). Although this comprehensive review of nutrition‐specific interventions exists for children under 5 years, no review has yet considered the combined evidence from nutrition‐specific and nutrition‐sensitive interventions during the complementary feeding period (6–23 months of age), which is of critical importance for the promotion of optimal growth, health and development. Furthermore, there has been no specific focus on characterising diet quality during the complementary feeding period; this early life risk factor influences childhood growth, and is a pathway to the development of chronic diseases in later life (Lynch & Davey‐Smith, 2005).

It is important to develop interventions that align with the current priorities of local, national and international stakeholders in relation to IYCF programmes, alongside gathering evidence from intervention studies to address IYC undernutrition. Combining evidence‐based research with multisectoral policy and planning with stakeholders to identify platforms for the delivery of community‐based interventions is more likely to lead to successful and sustainable health improvements for IYC (Bhutta et al., 2013). Indeed, engagement across different sectors and stakeholders is recognised by the WHO (2018) as an important approach to help achieve a 40% reduction in stunting in IYC under 5 years by 2025.

In summary, given the rapid urbanisation in SSA and the high prevalence of undernutrition, the nutritional status of IYC living in urban low‐income informal settlements is an important area for improvement. However, such improvements require evidence regarding the likely success of nutrition‐related interventions in the most vulnerable age group of IYC transitioning to complementary feeding in urban poor environments, as well as input from stakeholders to identify their priority areas for intervention. Therefore, we aimed to (a) systematically review current evidence in a rapid review of nutrition‐specific and nutrition‐sensitive interventions for improving the nutritional status of IYC aged 6–23 months living in urban poor areas (specifically low‐income informal settlements or slums) in LMICs and (b), through stakeholder engagement, identify the highest ranking evidence gaps for improving IYCF programmes and policies (focusing on complementary feeding) in children under 24 months in SSA.

## 2 METHODS

### 2.1 Rapid review

A rapid review approach, with a simplified systematic review process, was used to obtain context‐sensitive knowledge in a shortened timeframe (Grant & Booth, 2009; Khangura, Konnyu, Cushman, Grimshaw, & Moher, 2012). The rapid systematic review protocol was registered on PROSPERO (registration number: CRD42018091265). The search was originally conducted to review and synthesise evidence on nutrition‐sensitive and nutrition‐specific interventions to improve the nutritional status of IYC in urban low‐income informal settlements in SSA. After screening titles, abstracts and full texts for inclusion (completed in May 2018), this search yielded one eligible article. Following consultation with stakeholders (government, academic and NGO representatives) at a meeting held in Nairobi, Kenya, in June 2018, the protocol was modified by expanding the search to include all LMICs because of the lack of evidence in SSA. The revised protocol was updated and reregistered with PROSPERO (registration number as above) in June 2018. For this review, LMICs were defined using the World Bank Group (2019) classification scheme (based on gross national income per capita). The rapid review was designed to follow on from a scoping review from some of the authors that examined risk factors for malnutrition in slums and the impact of interventions on children's health in LMICs (Goudet, Griffiths, Bogin, & Madise, 2017). Key differences between the current protocol and the previous scoping review were to (a) include nutrition‐sensitive as well as nutrition‐specific interventions; (b) focus on IYC aged 6–23 months as opposed to all children under 5 years; (c) focus on diet quality outcomes, in addition to anthropometric outcomes. The search dates were set to identify new studies published since the scoping review search was completed in December 2013 (Goudet et al., 2017).

#### 2.1.1 Inclusion/exclusion criteria

Inclusion criteria for studies were based on the Population, Intervention, Comparison(s) and Outcome (PICO) structure (Thomas, Kneale, McKenzie, Brennan, & Bhaumik, 2019): (a) IYC aged 6–23 months in urban low‐income informal settlements or slums in LMICs; (b) nutrition‐specific or nutrition‐sensitive interventions, according to the definitions outlined by Ruel et al. (2013); and (c) quantitative outcomes of nutritional status indicators (length‐for‐age [LAZ], weight‐for‐height [WHZ] and weight‐for‐age [WAZ] *z*‐scores), undernutrition (stunting, underweight, wasting and micronutrient deficiencies) and diet quality (i.e., infant and young child MDD score, MMF and MAD). Eligibility criteria for the year of publication were from January 2014 to July 2018 (date of the search completion). The search, and summary of included studies, was completed prior to the stakeholder consultation (Delphi process) to inform this aspect of the research.

We included studies that specified the location as being a slum or low‐income informal settlement in accordance with the UN‐Habitat (2004) definition criteria, that is, lacking one or more of the following indicators: access to improved water and sanitation facilities, security of tenure, durability of housing and sufficient living area. When the location of a study was not clearly defined in a published paper, we consulted team members/collaborators who had local knowledge and/or original authors to help us to decide if a study met our requirements for inclusion as an urban low‐income informal settlement or slum.

#### 2.1.2 Search strategy

Four electronic databases were searched: Medline, Scopus, Web of Science and Embase. An example search strategy (Medline) and the list of LMICs included in the search are provided in Data S1. The reference lists of existing systematic and/or literature reviews were also screened to identify further studies for inclusion. Citation follow‐up and hand searches were used to identify studies up until January 2019, which yielded one additional study (Smuts et al., 2019).

#### 2.1.3 Screening, data extraction and analysis

##### Screening

Duplicates were removed before screening. The title, abstract and—subsequently—full‐text articles were screened against the inclusion criteria (by EKR, OM, TAZ, JMNC and EK). The review team pretested the screening form with an initial pilot phase of 15 studies. This process improved clarity of the inclusion criteria and consistency among assessors. An independent reviewer (RP) checked a random 10% sample of the excluded records at the title and abstract stages. Full‐text screening of articles was conducted by a second reviewer (EKR or OM) for confirmation. Any disagreements on study inclusion were discussed to reach consensus. If this was not possible, a third reviewer was consulted (PLG), and if necessary, authors were also contacted for further information about the study design to assess whether inclusion criteria were met.

##### Data extraction

A data extraction form was developed, piloted on two studies and then used to collate the data from all included studies. Information extracted from studies included title, author(s), year of publication, type of intervention (nutrition‐sensitive or nutrition‐specific), details of intervention (type of intervention/supplement, etc.), intervention duration, details of intervention compliance, outcome measures assessed (child nutritional status and nutritional quality of diet, anthropometrics), any other outcome measures related to nutritional status/diet quality/malnutrition (specifically, stunting, underweight, wasting and micronutrient deficiencies), detailed results (list all relevant outcome measures separately), study limitations, and lessons learned in delivering the intervention. Data were extracted by two independent reviewers (NP and OM) and checked by EKR.

##### Quality assessment process

Two reviewers (MM and EH) independently assessed the quality of the included studies using a 14‐item rating tool developed by Kmet et al. (2004). Their ratings were compared, and they came to an agreed rating for each criterion. As gold‐standard Cochrane guidance advises against the use of a rating score (Higgins & Green, 2006), a modified assessment classification was used. Instead of rating each criterion as either 0, 1 or 2, a qualitative, colour‐coded assessment of low quality/red (high risk of bias), medium quality/yellow or high quality/green (low risk of bias) was used, as has been done successfully in previous research (e.g., Rousham et al., 2020). If all 14 criteria were rated as ‘high/green’ then a ‘high’ overall quality assessment was awarded. Papers that had a combination of green and yellow (high/medium quality) ratings received a ‘medium’ overall quality rating. A ‘low’ overall quality assessment was given if any of the 14 criteria were assessed as ‘low/red’.

### 2.2 Stakeholder consultation

We built on our findings from the rapid review by conducting a stakeholder consultation to determine the priority areas for improving IYCF programmes and policies in SSA. We adopted a consensus‐gathering approach based on the Delphi method (Iqbal & Pipon‐Young, 2009) and consulted a range of stakeholders (‘panellists’) who contributed to three phases of information generation and consensus gathering. Consultation methods included two face‐to‐face stakeholder workshops (in Nairobi, Kenya, and Lilongwe, Malawi) and a survey that was distributed either online, as a paper‐based survey or via individual telephone interviews with stakeholders. This project came about through a nutrition network involving two Africa‐wide organisations (African Population and Health Research Centre [APHRC], Kenya, and African Institute for Development Policy [AFIDEP], Malawi) and so these locations in Kenya and Malawi were the starting point for the stakeholder engagement activities, which expanded from there outwards to bring in stakeholders working across SSA.

#### 2.2.1 Participants and ethical approval

Participants were individuals with experience of working or conducting research into IYC nutrition in SSA. Purposive sampling was used to identify potential participants. Participants were all aged 18 years or over. Prior to recruitment, ethical clearance was obtained from Loughborough University, from the Amref Health Africa Ethics and Scientific Review Committee (AMREF‐ESRC P528/2018) in Kenya and from the National Committee on Research in the Social Sciences and Humanities (No. P.12/18/336) in Malawi. Informed consent was given by all participants who took part in the workshops or completed the survey.

#### 2.2.2 Delphi consensus‐gathering procedure

A modified Delphi method was implemented, comprising three rounds. The Delphi method is interested in the formation or exploration of consensus, and it is exceedingly useful for topic areas where there is limited research because input and ideas come from a range of expert stakeholders (Iqbal & Pipon‐Young, 2009). Initially, evidence from the rapid review's synthesis of the recent research evidence identified gaps in knowledge in relation to IYCF, particularly complementary feeding, in LMICs. The research team drew on their knowledge and experience to summarise the research gaps in relation to SSA. These evidence‐informed gaps formed the basis of the first round of the modified Delphi approach.

##### Round 1

Evidence gaps were shared with a range of stakeholders from Kenya and Malawi at a face‐to‐face meeting in Nairobi, Kenya, in June 2018 (*n* = 18). Stakeholders were identified by co‐investigators in each country based on a list of target sectors (e.g., Ministry of Health, NGOs including practitioners and implementers, policymakers, academics, county government health officials, research institutions, professional networks [e.g., the African Nutrition Society and UNICEF]) to ensure a breadth of views would be represented. These stakeholders were invited to contribute their views about whether these evidence gaps were deemed to be (the most) important, as well as to offer their perspective—and to generate ideas—on where more evidence is needed to improve the nutritional status of IYC living in urban low‐income informal settlements or slums in SSA. Stakeholders discussed the questions posed in small groups, facilitated by a discussion leader. Notetakers recorded stakeholders' views and opinions. An example question asked of the stakeholders was *What is the evidence needed to enable the formulation and implementation of policies and programmes to improve child nutritional status?* The use of open‐ended questions, yielding qualitative data, is recommended for Round 1 because at this stage, the Delphi method focuses on the ‘future thinking’ on the issue (Iqbal & Pipon‐Young, 2009). Notes from the discussions were subsequently used to inform Round 2.

##### Round 2

Following the stakeholder meeting (Round 1), the information gained was used to create a survey, which was developed and refined by the multidisciplinary research team. An initial pool of survey items was developed, reviewed and refined by the research team. The final survey comprised 57 items, alongside some questions about participants' background to give context on their level of experience. The survey items were divided into three sections: (A) Nutritional status of urban poor families (11 items); (B) What is already known? (18 items); and, (C) What future work is needed, and how can this be done? (28 items). At the start of each section, participants were reminded of the statements related to the nutritional status of urban poor families and programmes/interventions/policies that might exist to support IYC feeding or nutrition and to think about IYC under 2 years when responding. Participants were asked to indicate the extent to which they agreed/disagreed with each statement, using a 5‐point scale. The survey was either completed online or administered as a face‐to‐face interview with a wide range of stakeholders who worked across SSA to gain their input into the evidence gaps and where further work/research is required. The online survey was distributed via the African Nutrition Society and by sending emails directly to relevant stakeholders. The aim of this round was to determine whether there was broad consensus in respondents regarding the evidence gaps highlighted by the rapid review.

#### 2.2.3 Round 3

The third and final round was conducted (a) at a second stakeholder meeting held in Malawi in January 2019 and (b) by emailing all of the other respondents (i.e., those not in attendance at the meeting) who had completed the Round 2 survey. Synthesised views from Round 2 were shared with stakeholders for any comments. Stakeholders were asked to select priority areas from the list of future directions. Six items related to what future work is needed, and how this can be done, when thinking about the *causes* of (mal)nutrition in urban poor IYC (focusing on undernutrition), and 22 items related to what future work is needed, and how this can be done, thinking about *potential solutions* to (mal)nutrition in urban poor IYC (Data S2). Respondents were asked to indicate their top two priorities from the list of six items and their top seven from the list of 22 items. Responses were collated and used to determine what stakeholders working in SSA believed were the most important priority areas for future work.

#### 2.2.4 Data analysis

Descriptive statistics were calculated. Round 1 information was collated, synthesised and used to inform the generation of Round 2 questions. Consensus in Round 2 was defined as ≥70% of participants either agreeing/strongly agreeing or disagreeing/strongly disagreeing with each statement. The level of agreement was set at ≥70%, as in previous studies (e.g., Diamond et al., 2014; Vogel et al., 2019). All ‘neutral’ responses were removed prior to calculating the percentage agreement/disagreement to ensure that only those who were confident with their answer were included (as per Vogel et al., 2019). For Round 3, priority areas for future work were identified, and responses were summed across participants to indicate the items with the greatest support.

## 3 RESULTS

### 3.1 Rapid review

**FIGURE 1:**
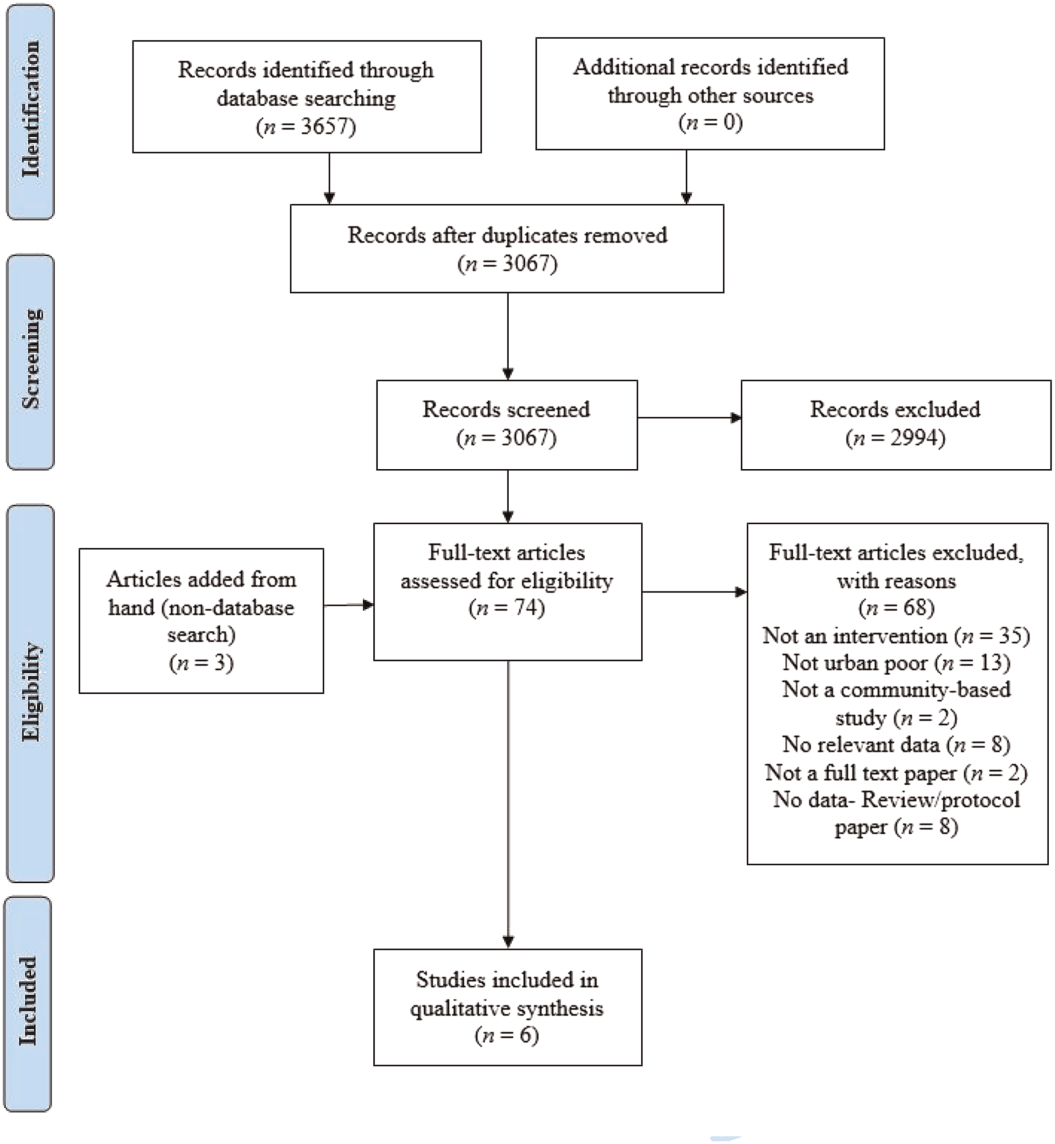
Flow chart highlighting rapid review search strategy and article inclusion

The search yielded 3,657 records. After removal of duplicates, 3,067 titles and abstracts were screened (Figure 1). Of these, 71 records qualified for full‐text screening, and 68 records were excluded at the full‐text stage. The three remaining records, plus three additional articles identified from reviewing reference lists or journal hand (non‐database) searches, met the inclusion criteria and were included in the review.

Our search also yielded three published protocol papers linked to ongoing intervention studies, one of which met our inclusion criteria. The study of Islam et al. (2018) aims to evaluate the effect of various doses, forms and frequencies of preventative daily zinc supplementation over a 24‐week period on reducing diarrhoea and improving growth, specifically LAZ, in IYC aged 9–11 months in the peri‐urban low‐income area of Mirpur (Dhaka, Bangladesh) (Clinical trials.gov: NCT03406793).

#### 3.1.1 Included studies

The six eligible studies included four randomised controlled trials (Iannotti et al., 2014; Martinez et al., 2018; Smuts et al., 2019; Strand et al., 2015) and two cluster‐randomised controlled trials (More et al., 2017; Tomlinson, Rotheram‐Borus, Scheffler, & le Roux, 2018).

#### 3.1.2 Description of studies

Of the six studies, two studies were carried out in India (More et al., 2017; Strand et al., 2015), one in Haiti (Iannotti et al., 2014), two in South Africa (Smuts et al., 2019; Tomlinson et al., 2018) and one in Bolivia (Martinez et al., 2018). Three studies involved nutritional supplements: one using vitamin B12 and folic acid supplements (Strand et al., 2015) and the two others administered daily lipid‐based nutrient supplements (LNSs; Iannotti et al., 2014; Smuts et al., 2019). Two studies involved home‐based visits: one included promotion of optimal IYCF practices by trained ‘Mentor Mother’ community health workers (Tomlinson et al., 2018) and one incorporated play‐based nutritional education messages (Martinez et al., 2018). Only one study took an integrated community‐based approach with the introduction of local resource centres in addition to home visits and day‐care centre interventions (More et al., 2017). The primary outcomes of More and colleagues' study were broad, including uptake of family planning/contraception and uptake of child immunisation, as well as treatment of severe acute malnutrition. In Tomlinson et al.'s (2018) study, home visits were conducted as part of an intervention to provide perinatal home visits to women with antenatal depression, so child development and nutritional indicators were not the primary outcomes of the intervention. Further details of the interventions are given in Table 1.

**TABLE 1:** Summary of a rapid review of intervention studies to improve nutritional status of infants and young children living in urban low‐income informal settlements in low‐ and middle‐income countries

First author, year; country/setting	Sample size (study completers)	Study design	Type and duration of intervention	Intervention details
● More et al., 2017, ^a^ ● Informal settlements in Mumbai, India	Intervention group: *n* = 5,371 children Control group: *n* = 5,180 children	Open‐labelled, cluster‐randomised controlled parallel trial	24‐month combined nutrition‐specific and nutrition‐sensitive intervention in children <5 years at baseline	*n* = 20 clusters were randomly allocated to have a resource centre (intervention group) and *n* = 20 clusters had no centre (control group). Each intervention group had a Society for Nutrition, Education and Health Action resource centre, which used approaches including home visits, organised group meetings, day care for malnourished children, community events, service provision and liaising with existing systems. Community intervention centres had a communication emphasis on maternal and neonatal health, child health and nutrition, sexual and reproductive health and prevention of violence against women. Integrated activities included home visits, group meetings, day care, community events, service provision and liaison.
● Strand et al., 2015 ● Urban neighbourhoods in Tigri and Dakshinpuri areas in New Delhi, India	Placebo group: *n* = 247 Vitamin B‐12 group: *n* = 250 Folic acid group: *n* = 249 Vitamin B‐12 and folic acid group: *n* = 247	Double‐blinded, randomised, controlled parallel trial	6‐month nutrition‐specific intervention in IYC aged 6‐ to 30‐months at baseline	Participants were randomly allocated to receive a placebo, folic acid and/or vitamin B‐12 lipid‐based paste. Supplements contained: Twice the RDA of vitamin B12 and/or twice the RDA of folic acid daily for 6 months. The vehicle for the supplementation was a lipid‐based paste, which contained 54.1 kcal, 0.71 g protein, 3.31 g fat per 10 g. IYC 6–11 months and ≥12 months were supplemented with 5 g/day (one teaspoon) and 10 g/day paste (two teaspoons), respectively. The placebo and vitamin supplements were matched in appearance and taste.
● Tomlinson et al., 2018 ● Townships—formal homes and informal shacks in Cape Town, South Africa	Intervention group: *n* = 231 Standard care group: *n* = 178	Open‐labelled, cluster‐randomised controlled parallel trial	18‐month post birth nutrition‐sensitive intervention	Neighbourhoods were randomly assigned to either: (a) SC (control group), which provided routine antenatal care or (b) PIP, which included SC and home visits by trained ‘Mentor mother’ community health workers. PIP mentor mothers had a communication emphasis on three outcomes during their household visits (≥8 visits per enrolled woman): 1) To use 'Prevent Mother‐to‐child Transmission' tasks for mothers living with HIV (MLH); to encourage MLHs to disclose HIV status to their partners; to encourage HIV testing and the use of condoms. 2) To reduce alcohol use/abuse among pregnant mothers (an adapted, one‐meeting evidence‐based intervention) 3) To encourage mothers to breastfeed for ≥6 months and to promote healthy food choices for optimal child growth and nutrition.
● Iannotti et al., 2014 ● Urban slums in Cap Haitien, Haiti	Control group: *n* = 144 3‐month LNS group: *n* = 126 6‐month LNS group: *n* = 150	Open‐labelled, randomised controlled parallel trial	3‐month to 6‐month nutrition‐specific intervention in singleton infants aged 6–11 months at baseline	Participants were randomly assigned to (a) a control group; (b) a 3‐month LNS; or (c) a 6‐month LNS (1:1:1 for group assignments) The LNS (20 g/day) provided 108 kcal, protein, fat and 19 vitamins and minerals, including vitamin A (400 μg), vitamin B‐12 (0.5 μg), iron (9 mg) and zinc (4 mg) at ≥80% of the recommended amounts. Mothers in LNS groups were instructed to offer the child one‐half of the LNS sachet in the morning and one‐half of the sachet in the afternoon. Instructions to mothers included washing hands and the sachet before feeding the LNS to their infant. They were also informed that the LNS should not be used as replacement for other foods. All groups received messages about appropriate complementary feeding and hygiene practices.
● Martinez et al., 2018 ● Low‐income households in the 8th district of the city of El Alto, Bolivia	Intervention group: *n* = 786 Control group: *n* = 781	Open‐labelled, randomised controlled parallel trial	≤30‐month nutrition‐specific intervention in infants <12 months at baseline	Eligible households were randomly assigned to intervention and control groups (1:1 for group assignments). Trained community health workers delivered nutrition messages through home‐based participatory play with a focus on improving IYCF practices. During home visits (60–90 min; 1–2 per month), the age‐specific educational paths emphasised one message from each of the following three areas: Exclusive breastfeeding or complementary feeding, responsive feeding behaviour and hygiene (with an emphasis on handwashing). Regardless of start age, children graduated from the programme at 24 months. Participants did not receive supplements, complementary foods or health service provision as part of the programme.
● Smuts et al., 2019 ● Peri‐urban Jouberton area of the Matlosana municipality, north‐west province of South Africa	SQ‐LNS group: *n* = 151 SQ‐LNS‐plus group: *n* = 162 No‐supplement (control) group: *n* = 201	Partially blinded, randomised controlled parallel trial	6‐month nutrition‐specific intervention in children aged 6 months at baseline	Infants were randomly allocated to one of two intervention groups (SQ‐LNS/SQ‐LNS‐plus) or a no‐supplement (control) group (1:1:1 for group assignments). SQ‐LNS/SQ‐LNS‐plus groups (20 g/day): Parents were asked to incorporate 1 × 20 g sachet daily into their child's first meal, mixed into their usual maize‐based complementary diet. The SQ‐LNS (50% of RNI for most micronutrients) provided 114 kcal, protein, fat, linoleic acid, α‐linolenic acid, and 20 micronutrients. The SQ‐LNS plus contained more protein (from skim‐milk powder) and choline (from lecithin), vitamin C and E than the SQ‐LNS supplement and 4 additional micronutrients (phosphorus, potassium, magnesium, manganese), DHA, arachidonic acid, l‐lysine and phytase. All groups were treated and monitored similarly, but the control group did not receive supplements. Participants and study staff involved in supply of the two SQ‐LNS products were blinded for the version of the SQ‐LNS that intervention participants received.

Abbreviations: DHA, docosahexaenoic acid; IYCF, infant and young child feeding; LNS, lipid‐based nutrient supplement; PIP, The Philani Intervention Program; RDA, recommended daily allowance; SC, standard care; SQ‐LNS, small‐quantity lipid‐based nutrient supplement.

^a^ Data presented are from a subcohort of IYC aged 6–23 months, with permission from UCL Institute for Global Health and Society for Nutrition, Education and Health Action (SNEHA).

#### 3.1.3 Quality assessment summary

All six studies achieved an overall quality rating of medium as they all contained a combination of green and yellow ratings across the 14 criteria outlined by Kmet et al. (2004). This indicates that these studies are relatively reliable sources of evidence with a fairly low risk of bias.

#### 3.1.4 Outcomes of the research studies included in the rapid review

The outcomes, process outcomes/participation rates, compliance details, study limitations and lessons learned from the intervention studies included in this review are summarised in Table 2. Strand et al. (2015) found that children who received vitamin B12 for 6 months had a significant increase in mean WAZ (0.07 *z*‐score, 95% confidence interval [CI] [0.01, 0.13], *p* < .05), compared with the placebo group. When using a subgroup analysis, vitamin B12 supplementation improved WAZ and LAZ in wasted, underweight and stunted children (significant baseline subgrouping variable × intervention interaction; *p* < .05).

**TABLE 2:** Summary of outcomes, study limitations and lessons learned from a rapid review of interventions studies to improve nutritional status of infants and young children living in urban low‐income settlements in low‐ and middle‐income countries

First author, year; country	Relevant outcomes measured	Detailed results (relevant outcomes)	Intervention compliance	Study limitations	Lessons learned in delivering the intervention
● More et al., 2017, ^a^ ● Mumbai, India	Stunting, underweight, wasting, micronutrient deficiencies, MAD, MDD	For IYC aged 6–23 months, the community intervention did not have a significant effect on underweight (OR 0.840, 95% CI [0.621, 1.136], *p* = .257) and stunting (OR 1.231, 95% CI [0.918, 1.651], *p* = .166); and there was a modest effect on wasting after controlling for the age of the child (OR 0.657, 95% CI [0.430, 1.004], *p* = .052). Following the intervention, achieving MDD and MAD in IYC aged 6–23 months increased, although not significantly, in the intervention group compared with the control group (OR 1·54, 95% CI [0·99, 2·39] and 1·58, 95% CI [0·94, 2·65], respectively). This was a community‐level intervention involving a census of eligible participants before and after the intervention. The population turnover was estimated to be 30% annually.	Not applicable.	The impact of the intervention on anthropometric wasting (WHZ) was limited; this may have been linked to movement of children into the clusters without full immunisation. Government schemes and the activities of municipal and nongovernmental providers may have improved health in control clusters, but authors found no evidence of contamination.	A community resource centre trial helped to increase MAD in IYC. Informal settlements have different cultural, structural and legal statuses from formal settlements. The range of issues that community organisers had to address challenged their ability to focus on primary outcome measures. Future trials could consider prioritising activities that address risk versus general activities, for example, the frequency of growth monitoring could be decreased in IYC who are meeting growth standards.
● Strand et al., 2015 ● New Delhi, India	Stunting, underweight, wasting, plasma vitamin B‐12, Folate, and total homocysteine (marker of folate and vitamin B‐12) status	The vitamin B‐12 group increased their mean WAZ by 0.07 *z*‐score, 95% CI [0.01, 0.13], *p* < .05). WAZ and LAZ increased significantly after vitamin B‐12 supplementation in wasted, underweight and stunted children (*p* < .05). The interventions had no impact on other anthropometric indicators. The geometric mean vitamin B‐12 concentration was 1.28 pmol/L, 95% CI [1.14, 1.44] times higher in those who were given vitamin B‐12 supplements, compared with those not assigned to this supplement. The geometric mean folate concentration was 3.14 nmol/L, 95% CI [2.56, 3.86] times higher in those who were assigned to folic acid supplementation. Both supplementations resulted in a decreased tHcy concentration with geometric mean ratios of 0.78 μmol/L, 95% CI [0.72, 0.83] and 0.83, 95% CI [0.77, 0.89], respectively. The loss to follow up among randomly assigned IYC was negligible (<1%).	96% of scheduled doses reportedly consumed.	The lack of a significant overall effect of the intervention on linear growth may be due to a combination of low supplement doses and the short exposure time.	Studies are needed to estimate the relative importance of several modifiable risk factors for suboptimal growth, including access to WASH, immunisation, treatment of infections, maternal education and nutritional deficiencies. These studies should consider the degree to which suboptimal folate and vitamin B‐12 status contributes to this multifaceted scenario. Future studies should consider higher doses and longer supplementation times (>6 months).
● Tomlinson et al., 2018; ● Cape Town, South Africa	Stunting, underweight	PIP infants at 18 months of mothers with antenatally depressed mood were significantly more likely to have WAZ ≥ −2 (OR 4.37, 95% CI [1.03, 18.49], compared to SC. The interventions had no impact on other anthropometric indicators. At 18 months, the attrition rate was 16% for both control and intervention groups. The study does not provide information as to whether it considered attrition rate while computing the required sample size.	Not reported.	The authors relied on a screening tool instead of a full diagnostic interview for depression.	Health and developmental outcomes among infants born to mothers with antenatally depressed mood were improved by mentor mother home visitor programme, which did not have a specific focus on maternal mental health. Further generalist community health worker‐delivered home visiting interventions are needed to investigate whether active screening for, and treatment of, antenatal d epression could have further benefits on child growth and cognitive functioning.
● Iannotti et al., 2014 ● Cap Haitien, Haiti	Stunting, wasting	6‐month LNS supplementation significantly increased mean LAZ (±SE) by 0.13 ± 0.05 *z*‐score and the WAZ by 0.12 ± 0.02 *z*‐score, compared with the control group after adjustment for child age (both *p* = .02). Compared with the control, the effects of 6‐month LNS were sustained 6 months post‐intervention for LAZ: 0.10 ± 0.05 *z*‐score (*p* = .04) and WAZ: 0.11 ± 0.04 *z*‐score (*p* = .02). 3‐month LNS supplementation had a negative effect on LAZ: −0.12 ± 0.05 *z*‐score (*p* = .03), which was sustained 6 months post‐intervention: −0.11 ± 0.05 *z*‐score (*p* = .03), compared with the control group. There was no impact of 3‐month LNS on WAZ: 0.01 ± 0.05 *z*‐score (*p* = .86), compared with the control group. 74.8% and 71.3% of the participants were followed up to 6 and 12 months, respectively. The sample size had factored a 25% attrition rate. Other than the 3‐month LNS group, which had an attrition rate of 35.7% at 6 months, loss to follow up for control and 6‐month LNS was within the assumed rate at 24.6% and 25.7%, respectively.	98% (3‐month LNS group) and 97% (6‐month LNS group) self‐reported consumption of monthly LNS. In both groups, 6% complied with the twice‐daily, split dose LNS consumption protocol.	Although adjustments were made for child age in the regression analyses, higher mean age was evident in the 6‐mo LNS group at baseline. This could have confounded the impact of LNS on LAZ, an outcome that is tends to be more influenced in younger IYC. There was a high loss to follow up due to the high mobility of the population (relocation was the main reason cited for drop out). In the 3‐month LNS group lost to follow‐up, the mean age of mothers was younger, and children showed a significantly greater WAZ (*p* < .05). By visit 5 of 7, children who remained in the trial from the 3‐month LNS group were younger than those in the control group (*p* < .05). Due to the nature of the intervention, it was not feasible to fully blind the allocation of participants.	Daily LNS improved linear growth in urban IYC, with sustained effects 6 months post‐intervention. Findings indicated that complementary interventions may be needed in combination with the LNS to address diarrhoea‐related morbidities and promote continued breastfeeding. The authors suggested that small‐quantity lipid may be a preferable strategy for improving micronutrient status and promoting healthy growth over longer extended supplementation periods, when compared to the use of ready‐to‐use supplemental food.
● Martinez et al., 2018 ● El Alto, Bolivia	Stunting, underweight, wasting, anaemia, MDD	There were no effects of the intervention on anthropometric indicators (WHZ, LAZ or WAZ) or anaemia, compared with the control (all *p* > .05). The recommended MDD (foods score ≥4) did not significantly increase in response to the intervention (mean difference of 0.003 in the proportion of children with foods score of at least four, 95% CI [−0.002, 0.028], *p* = .842]). However, MDD (foods score ≥5) increased in response to the intervention (mean difference of 0.046 in the proportion of children with foods score of at least five; 95% CI [0.004, 0.087], *p* = .032), compared with the control. By the end of the study, about 22% of the participants were lost to follow up, and this did not differ between the two study arms. The initial sampling took into consideration foreseen attrition. The authors conducted an ex post estimation of effect size using the final sample, which resulted in almost the same effect to the one assumed initially.	Households completing the intervention programme: 66% (study reporting); 73% (self‐reported). 6% of control households reported participating in the intervention.	A high drop‐out rate of ~21% was observed—this was balanced across treatment assignments. No data were presented on IYCF practices of the whole sample at baseline or the subsample of children recruited before birth (~1/5 of the sample).	The home‐based participatory play intervention is a promising delivery model to improve recommended IYCF practices. When combined with other interventions, behavioural change strategies may be considered an effective component of health policies; however, they may not be sufficient to improve anthropometric indicators in isolation. The model offers a novel delivery platform for additional interventions, for example, provision of fortified complementary foods or supplementation. Further studies in this area should consider whether the direct involvement of influential community leaders or family members might lead to increased impact and sustainability of nutrition‐related behavioural change. Longer term follow ups are necessary to assess whether the observed changes in nutrition practice are sustainable over time and translate into improvements in child growth and development outcomes.
● Smuts et al., 2019 ● North‐west province of South Africa	Stunting, wasting, iron deficiency, IDA	SQ‐LNS‐plus had a positive impact on LAZ at 8 months (mean difference: 0.11 *z*‐score; 95% CI [0.01, 0.22], *p* = .032) and 10 months (0.16 *z*‐score; 95% CI [0.04, 0.27], *p* = .008) but not at 12 months (0.09 *z*‐score; 95% CI [−0.02, 0.21], *p* = .115) but no effect on WAZ, when compared with the control. SQ‐LNS had no significant effect on LAZ or WAZ at 8, 10 or 12 months, compared with the control. SQ‐LNS and SQ‐LNS‐plus had a positive impact on iron deficiency (OR 0.41, 95% CI [0.22, 0.76], *p* = .004 and OR 0.26, 95% CI [0.14, 0.50], *p* < .001, respectively) and IDA (OR 0.11 95% CI [0.03, 0.37], *p* < .001 and OR 0.19; 95% CI [0.07, 0.48], *p* = .001, respectively) at 12 months, compared with the control. By the 12‐month visit, 31.5% of the recruited participants had dropped out of the study, which was more than the 25% assumed during sample estimation. The loss to follow up was highest in the intervention groups—SQ‐LNS at 39.6% and SQ‐LNS at 35.2% and lowest in the control group at 19.6%.	Proportion of days supplement consumed (based on empty sachets collected during weekly home visits): 94.1% and 94.4% in the SQ‐LNS and SQ‐LNS‐plus groups, respectively. Reported mean weekly consumption (based on a daily 4‐point pictorial scale): 78.8% and 78.2% in the SQ‐LNS and SQ‐LNS‐plus groups, respectively.	Results may have been confounded by the fact that: (a) drop‐out rate was higher in the two SQ‐LNS groups compared to the control group and (a) attrition was most common between 6 and 8 months. It was not feasible to fully blind the study due to the lack of placebo supplement in the control group.	The authors observed an early intervention effect (≤2 months) in linear growth in response to SQ‐LNS‐plus supplementation. This highlights the strength of regularly measuring anthropometric indicators, including length measurements. Further research should consider gathering context‐specific evidence, including optimal cost‐effectiveness, supplement composition, the most receptive age group and location of intervention.

Abbreviations: CI, confidence interval; IDA, iron deficiency anaemia; IYC, infants and young children; IYCF, infant and young child feeding; LAZ, length‐for‐age z score; LNS, lipid‐based nutrient supplement; MAD, minimum acceptable diet; MDD, minimum dietary diversity; OR, odds ratio; PIP, Philani Intervention Program; SC, standard care; SQ‐LNS, small‐quantity lipid‐based nutrient supplement; WASH, water, sanitation, and hygiene; WAZ, weight‐for‐age z score; WHZ, weight‐for‐height z score.

^a^ Data presented are from a sub‐cohort of IYC aged 6–23 months, with permission from UCL Institute for Global Health and SNEHA (Society for Nutrition, Education and Health Action).

Iannotti et al. (2014) found that intake of an LNS for 6 months was associated with a significant increase in LAZ (0.13 *z*‐score, 95% CI [0.08, 0.18], *p* = .02) and WAZ (0.12 *z*‐score, 95% CI [0.07, 0.17], *p* = .02), compared with the control group after adjustment for child age. The effects were sustained 6 months post‐intervention.

Smuts et al. (2019) assessed the impact of the daily consumption of two different small‐quantity LNS (SQ‐LNS) formulations on linear growth (primary outcome; for supplement details, see Table 1). Intake of SQ‐LNS‐plus for 6 months had a positive effect on combined LAZ when compared with the control condition (*p* = .036). This effect was driven by significant improvements in linear growth at age 8 months (0.11 *z*‐score, 95% CI [0.01, 0.22], *p* = .032) and 10 months (0.16 *z*‐score, 95% CI [0.04, 0.27], *p* = .008) but not at 12 months (0.09 *z*‐score, 95% CI [−0.02, 0.21], *p* = .115). No difference in the combined LAZ profile was evident between the SQ‐LNS and control conditions (*p* = .457). There was no significant effect of either SQ‐LNS or SQ‐LNS‐plus on WAZ (*p* = .559 and .186, respectively). Tomlinson et al. (2018) observed that infants' mothers with antenatal depression who were assigned to prenatal home visits as part of the Philani Intervention Program were significantly more likely to have a child who was not classed as underweight (WAZ ≥ −2) (odds ratio [OR] 4.37, 95% CI [1.03, 18.49], *p* < .05), compared to those assigned to the standard care group, although the CI for the estimate is very large casting some uncertainty on the findings.

Martinez et al.'s (2018) home‐based participatory play intervention had no significant effect on anthropometric indicators, or MDD, with 94% of IYC meeting MDD (defined as consuming at least four out of the seven key food groups during the previous 24 h [assessed via maternal recall]) in both treatment and control groups. However, IYC allocated to the treatment group did significantly increase their intake of five or more food groups (0.046 difference in the proportion of children with foods score of at least five food groups, 95% CI [0.004, 0.087]) versus the control group (*p* = .032).

More et al. (2017) published data on anthropometric indicators in children <5 years. The authors permitted us to conduct subgroup analysis, which enabled us to examine the impact of the intervention on anthropometric measures among IYC aged 6–23 months, although this subgroup analysis is likely based on an underpowered design as the original study did not target this group specifically. Our logistic regression analyses indicated that there was no significant effect of the intervention on WAZ (OR 0.840, 95% CI [0.621, 1.136], *p* = .257), LAZ (OR 1.231, 95% CI [0.918, 1.651], *p* = .166) and WHZ (OR 0.657, 95% CI [0.430, 1.004], *p* = .052) in children aged 6–23 months. However, in their original paper, the authors illustrated that the treatment group had an increased likelihood of achieving MDD following the intervention compared with control (OR 1·54, 95% CI [0.99, 2·39]) (More et al., 2017).

Together, the rapid review results highlighted a lack of evidence from sub‐Saharan countries other than South Africa and that, even when broadening the search out to include LMICs, there is a dearth of evidence surrounding effective interventions for complementary feeding of urban poor children aged 6–23 months. The included studies focused primarily on anthropometric indicators and showed modest‐to‐no intervention effects on stunting or LAZ. Similarly, few studies reported outcomes related to complementary feeding practices or how nutrition‐sensitive factors impact on nutritional status in an urban poor context. Only two included studies were nutrition‐sensitive or combined nutrition‐specific and nutrition‐sensitive approaches (Tomlinson et al., 2018 and More et al., 2017, respectively). Our review results indicated that studies tended to focus on anthropometric outcomes rather than factors that lead to achievement of nutritional impact (e.g., complementary feeding practices). These findings informed phase two of our research and directly informed the stakeholder consultation (Delphi) process by helping to identify the evidence gaps around IYCF and complementary feeding in LMICs to inform the questions subsequently asked of the stakeholders.

### 3.2 Stakeholder consultation

#### 3.2.1 Descriptive statistics

Seventeen participants took part in Round 1 of the modified Delphi, 48 in Round 2 and 35 in Round 3. All participants had experience of working or conducting research into IYC nutrition in SSA but did not necessarily live in SSA. Participants in Round 2 provided background details. They were generally well educated: 23% (*n* = 11) held an undergraduate degree, 44% (*n* = 21) had a Masters (or equivalent) and 29% (*n* = 14) had a PhD. They worked in a range of occupations (e.g., nutritionists 54% [*n* = 26]; academics 17% [*n* = 8]; programme director/manager 10% [*n* = 5]). Participants reported experience working in a range of SSA countries (46% [*n* = 22] in Kenya; 25% [*n* = 12] in Malawi; 8% [*n* = 4] in Ghana, 8% [*n* = 4] in Nigeria; 2% [*n* = 1] in Ethiopia; 2% [*n* = 1] in South Africa; and 8% [*n* = 4] in more than one SSA country). Participants had a wide range of experience working in the field of IYC nutrition, ranging from 2 to 40 years (mean = 15 years; median = 14 years).

#### 3.2.2 Consensus testing

Consensus was reached on 47 (82.4%) of the 57 Round 2 items (see Table 3). For the 10 questions where consensus was not achieved, these items were initially reviewed and discussed by the authors. Based on this review and following consultation with stakeholders as part of Round 3, it was agreed that these items might never achieve consensus given the divergence of views across the different sectors, disciplines, occupations and geographical locations of participants.

**TABLE 3:** Results of Round 2 of the Delphi‐based consensus‐gathering approach from *n* = 48 stakeholders

	Agree/strongly agree (%)	Disagree/strongly disagree (%)
**Section A: Nutritional status of urban poor families (11 items)**		
1. There are existing intervention programmes that are aimed at improving the nutritional status of children in urban poor settings	82.5	17.5
2. There is monitoring and evaluation of such programmes	62.9	37.1
3. There is follow‐up of action plans from previous reviews/evaluations of programmes	53.1	46.9
4. There are organisations that have done work into infant and young child feeding (IYCF) in urban poor settings	94.7	5.3
5. There is sufficient documentation and stock‐taking of the on‐going IYCF work already being implemented in urban poor settings	34.4	65.6
6. IYCF messaging and nutritional counselling interventions are effective at increasing exclusive breast‐feeding	90.7	9.3
7. NUTRITION‐SPECIFIC interventions targeted at mothers who are feeding infants and young children in urban poor areas currently exist	80.5	19.5
8. NUTRITION‐SENSITIVE interventions targeted at mothers who are feeding infants and young children in urban poor areas currently exist	59.0	41.0
9. Policies and intervention programmes aimed at supporting infant and young child nutrition are NOT specific to the urban poor	90.9	9.1
10. To date, policies and intervention programmes aimed at supporting child nutrition have been effective at improving infant and young child nutrition for the urban poor	37.5	62.5
11. Policies and intervention programmes aimed at supporting infant and young child nutrition are already being implemented with the urban poor	70.0	30.0
**Section B: What is already known? (18 items)**		
12. The value of micronutrient supplementation in the context of maternal, infant and young child nutrition (MIYCN) is well understood for the urban poor	32.4	67.6
13. The need for exclusive breast‐feeding is well understood among urban poor caregivers	51.2	48.8
14. The value of exclusive breast‐feeding is well understood among urban poor caregivers	55.6	44.4
15. The recommendations regarding complementary feeding are well understood among urban poor caregivers (i.e., families understand what it is)	20.6	79.4
16. The value of complementary feeding is well understood among urban poor caregivers	24.2	75.8
17. The challenges experienced by urban poor families in relation to IYCF are well understood	20.5	79.5
18. Urban informal day‐care centres provide appropriate infant and young child feeding practices	0.0	100.0
19. Urban informal day‐care centres provide safe, hygienic environmental conditions	2.3	97.7
20. Poor quality of care (at home and/or in day‐care settings) plays a role in the poor nutritional outcomes among infants and children in urban poor settings	95.7	4.3
21. Urban poor caregivers require social support to help improve the nutritional outcomes of their children	100.0	0.0
22. Maternal education plays a role in the nutritional outcomes of children in urban poor settings	95.7	4.3
23. Inadequate maternal employment plays a role in the poor nutritional outcomes of children in urban poor settings	95.6	4.4
24. Lack of support plays a role in the poor nutritional outcomes of children in urban poor settings	97.8	2.2
25. Community health volunteers (CHVs) in urban poor settings lack the support needed to engage caregivers with child nutrition in their home environment	86.8	13.2
26. Poverty is a factor contributing to suboptimal practices in child feeding	93.0	7.0
27. Food insecurity is a factor contributing to suboptimal practices in child feeding	97.8	2.2
28. Lack of proper sanitation and hygiene contributes to infections among children, affecting their nutritional wellbeing	97.8	2.2
29. Lack of clean water and sanitation in urban poor areas impacts the nutritional status of young children	100.0	0.0
**Section C: What future work is needed, and how can this be done? (27 items)**		
30. The CAUSES of malnutrition in urban poor areas are not as well understood as in rural areas	65.9	34.1
31. More studies or evidence on the CAUSES of malnutrition in urban poor areas are needed	87.8	12.2
32. The reasons for poor complementary feeding in urban poor areas need to be better understood	90.0	10.0
33. There is a need for integrated, multisectoral analysis of existing country‐level data on NUTRITION‐SENSITIVE interventions programmes/policies	100.0	0.0
34. There is a need for integrated, multisectoral analysis of existing country‐level data on NUTRITION‐SPECIFIC interventions programmes/policies	100.0	0.0
35. Studies are needed to assess the cost effectiveness of NUTRITION‐SENSITIVE interventions in urban poor settings	100.0	0.0
36. Studies are needed to document the cost effectiveness of NUTRITION‐SPECIFIC interventions in urban poor settings	97.7	2.3
37. Evidence about access to food for the urban poor population needs to be generated	95.5	4.5
38. The urban poor community should be engaged to identify gaps and potential solutions	97.9	2.1
39. The evidence on IYCF that has been gathered by organisations to date should be used to evaluate the situation in urban poor areas	87.5	12.5
40a. There is a need for NUTRITION‐SPECIFIC interventions targeted at urban poor MOTHERS feeding infants and young children	92.7	7.3
40b. There is a need for NUTRITION‐SPECIFIC interventions targeted at urban poor ADOLESCENTS feeding infants and young children	95.5	4.5
41a. There is a need for NUTRITION‐SENSITIVE interventions targeted at urban poor MOTHERS feeding infants and young children	94.9	5.1
41b. There is a need for NUTRITION‐SENSITIVE interventions targeted at urban poor ADOLESCENTS feeding infants and young children	97.7	2.3
42. Efforts are needed to better understand the current practices around micronutrient supplementation in the context of maternal, infant, and young child nutrition (MIYCN)	97.7	2.3
43. Efforts are needed to better understand the current practices around exclusive breast‐feeding	95.3	4.7
44. Efforts are needed to better understand the current practices around complementary feeding	100.0	0.0
45. Empowering urban poor women by providing EDUCATION AND INFORMATION will enhance their decision‐making autonomy and improve the nutritional wellbeing of their children	95.3	4.7
46. Empowering urban poor women by facilitating SAVING AND INCOME GENERATION will enhance their decision‐making autonomy and improve the nutritional wellbeing of their children	97.9	2.1
47. Better quality day care is an important way to improve child nutrition in urban poor settings	97.8	2.2
48. The use of locally available foods will improve complementary feeding in urban poor settings	95.5	4.5
49. Other methods to change behaviour, besides information sharing, should be promoted to enhance optimal MIYCN in urban poor settings (e.g., use of visual aids; the care group model)	100.0	0.0
50. There should be a multisectoral approach to nutrition involving other sectors—e.g., WASH and agriculture	100.0	0.0
51. Complementary feeding programmes should be designed alongside agri‐nutrition	100.0	0.0
52. Specific policies regarding infant and young child nutrition are needed for urban poor settings	89.7	10.3
53. Messages around complementary feeding need to be tailored to respect the challenges faced by urban poor families	95.8	4.2
54. There is a need for policies specific to urban poor settings that are aimed at supporting child nutrition	94.9	5.1
55. Before any further programmes are developed or implemented, a situational analysis of the urban poor MUST be undertaken	95.3	4.7

##### Section A: Nutritional status of urban poor families (11 items)

This section had the greatest number of items for which consensus was *not* reached. Overall, respondents agreed with six items. These items related to agreement that IYC nutrition intervention programmes exist (e.g., *There are existing intervention programmes that are aimed at improving the nutritional status of children in urban poor settings*). There were five items where respondents did not agree. These related to stakeholders' perceptions surrounding a lack of monitoring/evaluation of existing intervention programmes, a lack of intervention work with urban poor families and a perceived lack of efficacy of existing programmes (e.g., *There is sufficient documentation and stocktaking of the on‐going IYCF work already being implemented in urban poor settings*). The lack of consensus likely reflects variability across the countries in which the stakeholders work in terms of the range of interventions offered for the urban poor, as well as the monitoring, evaluation and efficacy of these.

##### Section B: What is already known? (18 items)

There was a good deal of agreement achieved in response to these items with all but three items receiving consensus, two of which related to exclusive breastfeeding. Where there was a lack of consensus, it reflected varied breastfeeding practices and support across the countries in which participants worked. The third item asked whether the value of micronutrient supplementation in the context of maternal, infant and young child nutrition (MIYCN) is well understood for the urban poor, and while two‐thirds of participants (68%) disagreed/strongly disagreed with this statement, the lack of consensus reflects the heterogeneous contexts across countries/areas. Respondents tended to disagree/strongly disagree with five items that asked whether complementary feeding is well understood, whether challenges of urban poor families in relation to IYCF are well understood and whether urban informal day‐care centres provide safe, hygienic care for children, which indicates a need to generate evidence on these areas as a priority. Participants tended to agree/strongly agree with statements covering a variety of factors thought to impact on IYC nutrition (e.g., social support, maternal education, maternal employment, poverty, sanitation and hygiene), which suggests that these are well‐established risk factors and potential intervention targets.

##### Section C: What future work is needed, and how can this be done? (28 items)

Consensus (agreement) was achieved for all but one of the statements in Section C, which were a list of areas for future research and intervention. For research, this included work looking at integrated, multisectoral analysis of existing country‐level data on nutrition‐specific and nutrition‐sensitive intervention programmes/policies and on access to food for the urban poor population. Suggestions for future intervention included a need for both nutrition‐specific and nutrition‐sensitive interventions targeted at urban poor mothers feeding IYC, as well as the use of locally available foods to improve complementary feeding, and empowering urban poor women by providing education and information. There was a lack of consensus as to whether the *causes* of undernutrition in urban poor areas are as well understood as in rural areas; however, although not reaching the threshold for consensus, two‐thirds of participants (66%) agreed that the causes were less well understood in urban poor than rural areas.

#### 3.2.3 Identification of priority areas for future research and intervention (Round 3)

Given the wide range of Section C statements which achieved consensus in Round 2, these statements were divided into two groups (six items relating to what future work is needed linked to causes of IYC malnutrition and 22 items relating to what future work is needed linked to solutions to IYC malnutrition; Data S2). Round 3 asked each respondent to select two out of six statements and then seven out of 22 statements that they believe are the most important priority areas for future work. Thirty‐one individuals completed Round 3. The results suggest that, in relation to what future work is needed and how this can be done, ‘More studies or evidence on the CAUSES of malnutrition in urban poor areas are needed’ was the highest ranked priority area, with 16 of the 31 stakeholders (51.6%) selecting it (see Table 4). ‘Efforts are needed to better understand the current practices around complementary feeding’ was the next highest ranked, with 12 out of 31 stakeholders voting for this (38.7%) and ‘Evidence about access to food for the urban poor population needs to be generated’ was ranked third, receiving support from 11 of the 31 respondents (35.5%).

**TABLE 4:** Results of Round 3 of the Delphi‐based consensus‐gathering approach from *n* = 31 stakeholders to determine priority areas for future research (listed in priority order)

Statement	Number (%) of endorsements for this statement
31. More studies or evidence on the CAUSES of malnutrition in urban poor areas are needed	16 (52%)
44. Efforts are needed to better understand the current practices around complementary feeding	12 (39%)
37. Evidence about access to food for the urban poor population needs to be generated	11 (35%)
32. The reasons for poor complementary feeding in urban poor areas need to be better understood	9 (29%)
30. The CAUSES of malnutrition in urban poor areas are not as well understood as in rural areas	8 (26%)
43. Efforts are needed to better understand the current practices around exclusive breast‐feeding	6 (19%)

In relation to potential solutions to (mal)nutrition in urban poor IYC, two statements were joint highest ranked (see Table 5). These were ‘Before any further programmes are developed or implemented, a situational analysis of the urban poor MUST be undertaken’ and also ‘The urban poor community should be engaged to identify gaps and potential solutions’. Nineteen of the 31 stakeholders (61.3%) selected these. The third most favoured was ‘There is a need for integrated, multisectoral analysis of existing country‐level data on NUTRITION‐SENSITIVE interventions programmes/policies’, which was endorsed by 17 stakeholders (54.8%). Together, these choices highlight that there was the greatest consensus around a need for conducting a situational analysis and involving urban poor community members to determine their needs, as well as for conducting integrated, multisectoral analysis of existing nutrition‐sensitive interventions.

**TABLE 5:** Results of Round 3 of the Delphi‐based consensus‐gathering approach from *n* = 31 stakeholders to determine priority areas for future research (listed in priority order)

Statement	Number (%) of endorsements for this statement
55. Before any further programmes are developed or implemented, a situational analysis of the urban poor MUST be undertaken	19 (61%)
38. The urban poor community should be engaged to identify gaps and potential solutions	19 (61%)
33. There is a need for integrated, multisectoral analysis of existing country‐level data on NUTRITION‐SENSITIVE interventions programmes/policies	17 (55%)
45. Empowering urban poor women by providing EDUCATION AND INFORMATION will enhance their decision‐making autonomy and improve the nutritional wellbeing of their children	16 (52%)
47. Better quality day care is an important way to improve child nutrition in urban poor settings	15 (48%)
49. Other methods to change behaviour, besides information sharing, should be promoted to enhance optimal MIYCN in urban poor settings (e.g., use of visual aids; the care group model)	15 (48%)
35. Studies are needed to assess the cost effectiveness of NUTRITION‐SENSITIVE interventions in urban poor settings	12 (39%)
53. Messages around complementary feeding need to be tailored to respect the challenges faced by urban poor families	12 (39%)
48. The use of locally available foods will improve complementary feeding in urban poor settings	10 (32%)
40a. There is a need for NUTRITION‐SPECIFIC interventions targeted at urban poor MOTHERS feeding infants and young children	9 (29%)
50. There should be a multisectoral approach to nutrition involving other sectors—e.g., WASH and agriculture	9 (29%)
39. The evidence on IYCF that has been gathered by organisations to date should be used to evaluate the situation in urban poor areas	8 (26%)
41a. There is a need for NUTRITION‐SENSITIVE interventions targeted at urban poor MOTHERS feeding infants and young children	8 (26%)
34. There is a need for integrated, multisectoral analysis of existing country‐level data on NUTRITION‐SPECIFIC interventions programmes/policies	7 (23%)
46. Empowering urban poor women by facilitating SAVING AND INCOME GENERATION will enhance their decision‐making autonomy and improve the nutritional wellbeing of their children	7 (23%)
36. Studies are needed to document the cost effectiveness of NUTRITION‐SPECIFIC interventions in urban poor settings	6 (19%)
51. Complementary feeding programmes should be designed alongside agri‐nutrition	6 (19%)
40b. There is a need for NUTRITION‐SPECIFIC interventions targeted at urban poor ADOLESCENTS feeding infants and young children	5 (16%)
52. Specific policies regarding infant and young child nutrition are needed for urban poor settings	5 (16%)
41b. There is a need for NUTRITION‐SENSITIVE interventions targeted at urban poor ADOLESCENTS feeding infants and young children	4 (13%)
42. Efforts are needed to better understand the current practices around micronutrient supplementation in the context of maternal, infant, and young child nutrition (MIYCN)	3 (10%)
54. There is a need for policies specific to urban poor settings that are aimed at supporting child nutrition	3 (10%)

## 4 DISCUSSION

This research aimed to systematically review evidence on nutrition‐specific and nutrition‐sensitive interventions for improving the nutritional status of 6–23‐month‐old IYC living in urban poor areas and, also, to engage stakeholders and identify the highest ranking evidence gaps for improving IYCF programmes and policies. Both the findings from the rapid systematic review and the Delphi exercise point to a need for more evidence regarding complementary feeding, and the factors that drive it, in poor urban areas in LMICs, as well as highlight a need for additional interventions in LMICs to support optimal complementary feeding.

The rapid review identified six intervention studies published since 2014. The quality assessment indicated that these were all of medium quality. It is worth noting that reporting of outcome measures and details of controlling for confounds were the areas where studies commonly achieved lower (medium) quality ratings, which reflect the write‐up of the study rather than the quality of the research conducted. Although intervention adherence was high in most studies and indicators of maternal knowledge and IYC nutritional intake (e.g., dietary diversity) increased as a result of the interventions, the effects on IYC anthropometric status were small, even when statistically significant. No studies were identified that reported interventions to improve the complementary feeding of IYC from low‐income urban settings in SSA, other than one of the two studies in South Africa. It is noteworthy that only two studies assessed diet quality (Martinez et al., 2018; More et al., 2017). Most of the six studies focused on anthropometric outcomes rather than the pathways to achieving those outcomes, and no included studies specifically examined complementary feeding. Given the critical role of complementary feeding practices on the nutrition of 6–23‐month‐olds, future research should focus on this important component among the urban poor. Furthermore, the use of health service/community activities and home‐based participatory play interventions could also provide an important platform for improving IYCF practices (Martinez et al., 2018; More et al., 2017; Tomlinson et al., 2018). Martinez et al. (2018) noted that 6% of control group households reported taking part in the home‐based intervention study. Therefore, it is worth considering strategies to minimise treatment ‘contamination’ between intervention and control groups in future studies, such as adopting a cluster randomisation study design (Torgerson, 2001). Our rapid review shows that the gap in evidence around nutrition‐sensitive interventions in urban areas of low‐income countries persists, confirming the earlier findings of Goudet et al. (2017). There has been an increase in such studies in rural areas, such as the recent integrated WASH and young child nutrition interventions in Bangladesh, Kenya and Zimbabwe (Humphrey et al., 2019; Luby et al., 2018; Null et al., 2018), further highlighting the evidence gaps for nutrition‐sensitive interventions in low‐income urban communities. The evidence gaps were further endorsed by stakeholders participating in the Delphi consensus‐gathering exercise who identified ‘a need for integrated, multi‐sectoral analysis of existing country‐level data on nutrition‐sensitive interventions programs/policies’ as one of the top three priority areas for further work.

Stakeholders agreed that more work was required to better understand complementary feeding and the factors that are important in urban poor areas. They highlighted the need to involve local communities in identifying gaps and potential solutions for addressing undernutrition in informal settlements. The value of obtaining the views and opinions of communities in health‐promotion interventions is not a new idea (e.g., Cyril, Smith, Possamai‐Inesedy, & Renzaho, 2015), but there remains a lack of application of this approach for informing the development of suitable intervention strategies and content to improve undernutrition in poor informal urban areas in Africa. More et al. (2017) involved residents in community‐based voluntary activities while Tomlinson et al. (2018) employed a positive deviance approach by recruiting and training ‘mentor mothers’ from the local townships to deliver home visits. Better understanding the specific features and requirements of individuals raising children in urban poor areas is vital for ensuring that interventions are effective and sustainable. However, very few studies included in a recent Cochrane review addressed the urban poor context or considered intervention outcomes within this particular setting (Goudet et al., 2019). Using local knowledge could help to move interventions forward and improve success rates and efficacy. Engagement with local populations, coupled with a country‐level situational analysis, would be an important starting point for contextualising such interventions.

More than 75% of stakeholders across SSA countries reported that the recommendations around, and the value of, complementary feeding were not well understood by urban poor caregivers. Moreover, 90% believed that the reasons for poor complementary feeding in urban poor areas need to be better understood, and 100% of participating stakeholders indicated that efforts are needed to better understand current practices around complementary feeding. Together, these findings highlight that further work around complementary feeding in urban poor areas is a priority across SSA. They also reveal that existing evidence around complementary feeding in urban poor areas (e.g., Kimani‐Murage et al., 2011; Macharia, Ochola, Mutua and Kimani‐Murage, 2018) is not reaching key stakeholders, so improved dissemination of research findings to relevant stakeholders is another future priority. Furthermore, much of this existing evidence tends to be limited to very large poor urban areas in mostly middle‐income country contexts, which means that it is not necessarily so relevant to very different poor urban environments in low‐income settings, highlighting another gap to address in the future. In contrast to the need identified for further work around complementary feeding in urban poor areas, it was generally agreed (91%) that existing IYCF messaging and nutritional counselling interventions are effective at increasing exclusive breastfeeding, indicating that this is less of a priority area for nutrition‐related interventions. Empowering urban poor women by providing education and information was agreed by stakeholders as an important way to improve complementary feeding practices, and while such behaviour change practices are frequently used in interventions across LMICs, it is noteworthy that other aspects of behaviour change (e.g., the provision of social support or demonstration of a behaviour) have been shown to be effective in bringing about changes and might warrant inclusion alongside education in future intervention development (Webb Girard, Waugh, Sawyer, Golding, & Ramakrishnan, 2020).

The stakeholder exercise revealed consensus regarding the nutritional status of urban poor families; what is already known/not known about IYC nutrition; and what future work is needed, and how it can be done. Stakeholder opinions were more discordant in relation to the efficacy, monitoring, evaluation and documentation of existing programmes relating to IYCF in the urban poor. Given the varied job roles, experiences and geographical locations of participants, consensus around these items is less likely to be achieved. Therefore, when moving forward with this work, consideration will be given to between‐country differences in factors like documentation and stocktaking of the ongoing IYCF work already being implemented in urban poor settings.

Importantly, some clear priority areas were identified. Stakeholders prioritised conducting a situational analysis of the urban poor and doing this before any further programmes are developed and implemented. Over half of the stakeholders reported that more work surrounding the causes of undernutrition in urban poor areas would be a key area for future work. This could include consideration of the burden and nature of infectious disease morbidity and its contribution to childhood undernutrition as well as nutritional determinants. Stakeholders believed that urban poor community members should be engaged in identifying gaps and potential solutions. These results are hugely beneficial for driving the direction of future research in this area.

Strengths of this review include the systematic search and retrieval of articles from four databases, conducting additional analyses relevant to our aims, and the synthesis of evidence from both nutrition‐specific and nutrition‐sensitive interventions We used a rapid, rather than a systematic, review as we needed to complete a rapid evidence synthesis that balanced time constraints with considerations for bias. For this reason, we did not include a search of the grey literature or non‐English articles, nor did we aim to conduct a meta‐analysis, but we did conduct a quality assessment of the six studies. As the rapid evidence synthesis informed our stakeholder consultation (Delphi process), we limited the start of the search period based on the date of publication. Therefore, studies published before our search period (January 2014), including nutrition‐sensitive study elements (e.g., WASH) and the ones with diet quality outcomes, were outside the search parameters. For example, the study by Penny et al. (2005), which was included in the earlier review by Goudet et al., observed that an intervention to improve nutrition education delivered through Peruvian health services led to some improvements in complementary feeding practices (main secondary outcome) when compared with IYC from control areas. The Goudet et al. (2017) review focused on the measures of undernutrition rather than complementary feeding specifically, so this information is not captured either here or in the earlier review. The six included studies had a broad geographical spread but had high heterogeneity in terms of nutritional supplements/interventions, outcome indicators and follow‐up periods. Strengths of the Delphi study are the consultation of a wide range of stakeholders, the use of a three‐step process to garner consensus and the contribution of stakeholders from the outset. Limitations include unequal representation from across SSA countries and a lack of representation from all SSA countries, meaning that generalisability is somewhat limited and we were unable to conduct analysis by country. A further limitation was the absence of questions relating to IYC consumption of 'junk' foods and sugar‐sweetened beverages, given evidence that intake of these is increasing in many LMICs (e.g., Monteiro, Moubarac, Cannon, Ng, & Popkin, 2013; Pries, Filteau, & Ferguson, 2019), and this is something that warrants exploration in future.

In conclusion, together, these findings highlight the limited research into complementary feeding and the nutritional status of children aged 6–23 months in the urban poor context. There has been a dearth of studies regarding nutrition‐sensitive programme development and a lack of research on complementary feeding practices on the pathway to anthropometric outcomes in urban poor areas. Future studies are needed to address this knowledge gap. The rapid systematic review revealed a lack of interventions to improve nutritional status of IYC living in urban low‐income informal settlements in SSA and highlighted that effects were modest. When integrated with the Delphi findings, these results help to prioritise the next steps in terms of supporting the development of such interventions, namely, that more studies or evidence on the causes of undernutrition in urban poor areas are needed; a situation analysis of the urban poor needs to be undertaken; and the urban poor community should be engaged to identify gaps and potential solutions. These priority areas identified by the stakeholders provide a clear direction for future research and intervention to improve IYC nutrition in urban poor families in SSA.

## CONFLICTS OF INTEREST

The authors declare that they have no conflicts of interest.

## CONTRIBUTIONS

OM, EKR, MM, JMNC, RP, EK and PLG conducted the rapid review. EH, MM and JMNC led, and all authors contributed to, the stakeholder activities. MM, OM, EKR, PLG and EH drafted the paper. All authors contributed to critically revising the article and gave approval of the final version.

## Supporting information


**Data S1** Supporting Information

**Data S2** Supporting Information
