# Supplementary material for: Improving nutritional status among urban poor children in sub‐Saharan Africa: An evidence‐informed Delphi‐based consultation
Source: Matern Child Nutr. 2020 Nov 3;17(2):e13099. doi: 10.1111/mcn.13099 (PMC7988854; doi:10.1111/mcn.13099)
Supplement: Supplementary file 1 — Data S1 Supporting Information [file MCN-17-e13099-s001.docx]

**Supplementary file S1: Example search from Medline**

**Medline, Search all fields for:**

urban OR slum OR shanty OR ghetto OR shack OR bidonville OR squat OR informal settlement OR informal urban settlement OR taudi OR irregular settlement OR informal housing OR irregular settlement OR low income OR urban poor OR poverty

**AND**

nutrition OR undernourished OR malnutrition OR undernutrition OR wasting OR stunting OR stunted OR wasted OR kwashiorkor OR SAM OR GAM OR MAM OR growth falter OR low birth weight OR marasmus OR thin OR emaciated OR nutritional status OR nutrition OR malnutrition OR body mass index OR BMI OR short stature OR weight-for-age OR height-for-age OR Length-for-age OR weight-for-length OR MUAC OR mid upper arm circumference OR anthropometry OR skinfold thickness OR starvation OR underweight OR malnourishment OR dietary deficiency OR dietary diversity OR minimum acceptable diet OR diet quality OR hunger OR food deprived OR dietary energy requirement OR vitamin OR micronutrient OR ‘IYCN’ OR ‘Infant and young child nutrition’ OR ‘complementary food*’ OR ‘complementary feeding’ OR ‘supplement*’

**AND** child* OR infant* OR baby OR toddler* OR IYC OR ‘infant and young child’

**AND**

‘Low-income countr*’ OR middle-income countr*’ OR ‘LMIC*’ OR ‘less developed countr*’ OR ‘developing countr*’ OR ‘low and middle-income countr*’ OR ‘global south’ OR ‘developing nation’ OR ‘less economically developed’ OR

Afghanistan OR Guinea OR Rwanda OR Benin OR Guinea-Bissau OR Senegal OR Burkina Faso OR Haiti OR Sierra Leone OR Burundi OR Korea OR Somalia OR Central African Republic OR Liberia OR South Sudan OR Chad OR Madagascar OR Tanzania OR Comoros OR Malawi OR Togo OR Congo OR Mali OR Uganda OR Eritrea OR Mozambique OR Zimbabwe OR Ethiopia OR Nepal OR Gambia OR Niger OR Angola OR Indonesia OR Philippines OR Armenia OR Jordan OR São Tomé and Principe OR Bangladesh OR Kenya OR Solomon Islands OR Bhutan OR Kiribati Sri Lanka OR Bolivia OR Kosovo OR Sudan OR Cabo Verde OR Kyrgyz Republic OR Swaziland OR Cambodia OR Lao PDR OR Syria*c OR Cameroon OR Lesotho OR Tajikistan OR Congo OR Mauritania OR Timor-Leste OR Côte d'Ivoire OR Micronesia OR Tunisia OR Djibouti OR Moldova OR Ukraine OR Egypt OR Mongolia OR Uzbekistan OR El Salvador OR Morocco OR Vanuatu OR Georgia OR Myanmar OR Vietnam OR Ghana OR Nicaragua OR West Bank OR Gaza OR Guatemala OR Nigeria OR Yemen OR Honduras OR Pakistan OR Zambia OR India OR Papua New Guinea OR Albania OR Ecuador OR Algeria OR Fiji OR American Samoa OR Gabon OR Argentina OR Grenada OR Azerbaijan OR Guyana OR Belarus OR Iran OR Belize OR Iraq OR Bosnia and Herzegovina OR Jamaica OR Botswana OR Kazakhstan OR Brazil OR Lebanon OR Bulgaria OR Libya OR China OR Macedonia OR Colombia OR Malaysia OR Costa Rica OR Maldives OR Croatia OR Marshall Islands OR Cuba OR Mauritius OR Dominica OR Mexico OR Dominican Republic OR Montenegro OR Equatorial Guinea OR Namibia OR Nauru OR Panama OR Paraguay OR Peru OR Romania OR Russian Federation OR Samoa OR Serbia OR South Africa OR St. Lucia OR St. Vincent and the Grenadines OR Suriname OR Thailand OR Tonga OR Turkey OR Turkmenistan OR Tuvalu OR Venezuela

**Limited to:**

Publication date: from January 2014 to July 2018

Publication type: Journal articles

English language only
