# Supplementary material for: Improving nutritional status among urban poor children in sub‐Saharan Africa: An evidence‐informed Delphi‐based consultation
Source: Matern Child Nutr. 2020 Nov 3;17(2):e13099. doi: 10.1111/mcn.13099 (PMC7988854; doi:10.1111/mcn.13099)
Supplement: Supplementary file 2 — Data S2 Supporting Information [file MCN-17-e13099-s002.docx]

**Supplementary File S2: Stakeholder ranking results for priority areas for future work**

Stakeholders were asked to select the two statements, from the six below, that they thought were **the most important priority areas for future work**. These six items related to what future work is needed, and how this can be done, when thinking about **causes** of (mal)nutrition in urban poor IYC.

| **List of potential priority areas for future work: Causes of (mal)nutrition in urban poor IYC** | |
| --- | --- |
| 30 | The CAUSES of malnutrition in urban poor areas are not as well understood as in rural areas |
| 31 | More studies or evidence on the CAUSES of malnutrition in urban poor areas are needed |
| 32 | The reasons for poor complementary feeding in urban poor areas need to be better understood |
| 37 | Evidence about access to food for the urban poor population needs to be generated |
| 43 | Efforts are needed to better understand the current practices around exclusive breast-feeding |
| 44 | Efforts are needed to better understand the current practices around complementary feeding |

Next, stakeholders were asked to select seven statements from the list of 22 items below that they felt were **the most important priority areas for future work**. These 22 items related to what future work is needed, and how this can be done, thinking about **potential solutions** to (mal)nutrition in urban poor IYC.

| **List of potential priority areas for future work: potential solutions to (mal)nutrition in urban poor IYC** | |
| --- | --- |
| 33 | There is a need for integrated, multi-sectoral analysis of existing country-level data on NUTRITION-SENSITIVE interventions programs/policies |
| 34 | There is a need for integrated, multi-sectoral analysis of existing country-level data on NUTRITION-SPECIFIC interventions programs/policies |
| 35 | Studies are needed to assess the cost effectiveness of NUTRITION-SENSITIVE interventions in urban poor settings |
| 36 | Studies are needed to document the cost effectiveness of NUTRITION-SPECIFIC interventions in urban poor settings |
| 38 | The urban poor community should be engaged to identify gaps and potential solutions |
| 39 | The evidence on IYCF that has been gathered by organizations to date should be used to evaluate the situation in urban poor areas |
| 40a | There is a need for NUTRITION-SPECIFIC interventions targeted at urban poor MOTHERS feeding infants and young children |
| 40b | There is a need for NUTRITION-SPECIFIC interventions targeted at urban poor ADOLESCENTS feeding infants and young children |
| 41a | There is a need for NUTRITION-SENSITIVE interventions targeted at urban poor MOTHERS feeding infants and young children |
| 41b | There is a need for NUTRITION-SENSITIVE interventions targeted at urban poor ADOLESCENTS feeding infants and young children |
| 42 | Efforts are needed to better understand the current practices around micronutrient supplementation in the context of Maternal, Infant, and Young Child Nutrition (MIYCN) |
| 45 | Empowering urban poor women by providing EDUCATION AND INFORMATION will enhance their decision-making autonomy and improve the nutritional wellbeing of their children |
| 46 | Empowering urban poor women by facilitating SAVING AND INCOME GENERATION will enhance their decision-making autonomy and improve the nutritional wellbeing of their children |
| 47 | Better quality day care is an important way to improve child nutrition in urban poor settings |
| 48 | The use of locally available foods will improve complementary feeding in urban poor settings |
| 49 | Other methods to change behavior, besides information sharing, should be promoted to enhance optimal MIYCN in urban poor setting (e.g. use of visual aids; the care group model) |
| 50 | There should be a multi-sectorial approach to nutrition involving other sectors - e.g. WASH, agriculture |
| 51 | Complementary feeding programs should be designed alongside agri-nutrition |
| 52 | Specific policies regarding infant and young child nutrition are needed for urban poor settings |
| 53 | Messages around complementary feeding need to be tailored to respect the challenges faced by urban poor families |
| 54 | There is a need for policies specific to urban poor settings that are aimed at supporting child nutrition |
| 55 | Before any further programs are developed or implemented, a situational analysis of the urban poor MUST be undertaken |
